# Laboring Alone: Perinatal Outcomes during Childbirth without a Birth Partner or Other Companion during the COVID-19 Pandemic

**DOI:** 10.3390/ijerph20032614

**Published:** 2023-02-01

**Authors:** Antonín Pařízek, Petr Janků, Miloslava Kameníková, Petra Pařízková, Daniela Javornická, Dana Benešová, Vladimír Rogalewicz, Zdeněk Laštůvka, Miroslav Barták

**Affiliations:** 1 Department of Obstetrics and Gynecology, First Faculty of Medicine, Charles University and General University Hospital, 128 08 Prague, Czech Republic; 2 Department of Gynecology and Obstetrics, University Hospital Brno, 602 00 Brno, Czech Republic; 3 Department of Midwifery, Faculty of Health Sciences, Palacky University Olomouc, 779 00 Olomouc, Czech Republic; 4 Department of Biomedical Technology, Faculty of Biomedical Engineering, Czech Technical University in Prague, 272 01 Kladno, Czech Republic; 5 Department of Social Work, Faculty of Social and Economics Studies, J. E. Purkyně University, 400 96 Ústí nad Labem, Czech Republic

**Keywords:** birth companion, childbirth experiences, continuous childbirth support, women’s health, COVID-19

## Abstract

During the first wave of the COVID-19 pandemic in the spring of 2020, the government of the Czech Republic issued a nationwide ban on visitors to maternity wards. We studied whether the absence of a close person during labor due to this ban impacted perinatal indicators. This study was performed using an administrative observational questionnaire focused on absolute frequencies of events sent to maternity facilities across the Czech Republic. Completed answers were received from 33 facilities covering 4805 births during the study period in 2019 and 4514 births in 2020. The differences in individual parameters were tested using Pearson’s chi-squared homogeneity test. There were no significant differences between the two periods in spontaneous pre-term births (*p* = 0.522) or in the number of cesarean sections (*p* = 0.536). No significant changes were seen in either local or systemic analgesia. Data showed a significantly shorter (*p* = 0.026) first stage of labor in 2020 compared to 2019, while there was no significant difference (*p* = 0.673) in the second stage of labor. There was no statistically significant difference found for newborn perinatal adaptation. There were also no significant differences in intrapartum maternal injuries. Overall, we found no significant differences in basic perinatal indicators during the first wave of COVID-19 in 2020 compared to 2019. Although the absence of a close person may cause stress for the laboring women, it does not impair objective clinical outcomes.

## 1. Introduction

Childbirth in modern hospital settings is a highly standardized process, with stringent requirements put on the quality of obstetric care, with health care quality also emphasizing non-medical issues. In many countries, it is common that the child’s father or another close person can be present during the labor and delivery in any maternity facility [1]. Due to ethical considerations, it is normally impossible to perform studies on the contribution of a partner´s presence during childbirth. However, a significant benefit to expectant mothers´ well-being has been repeatedly mentioned [2,3]. The fathers benefit from the right to a two-week paid paternity leave based on their sickness insurance. They can start their paternity leave on any day they choose within six weeks from the child’s birth. This measure has also contributed to developing an environment where the father’s presence at childbirth is considered an important contribution to supporting family bonds between the mother, father, and child [4,5]. Based on our research, several studies were found on the presence of a close person at birth. However, none of the good-quality studies dealt explicitly with the absence of a companion at birth, as it has probably not been possible to construct such a study design due to ethical reasons.

In the spring of 2020, strict anti-COVID-19 measures were adopted in the Czech Republic that included a ban on the presence of any person, including fathers, in maternity facilities, which allowed us to study the impact of the father´s absence during childbirth on obstetric care outcomes. The authors stress the importance of the World Health Organization (WHO, Geneva, Switzerland) recommendations on intrapartum care for a positive childbirth experience, where the practical and emotional support from a birth companion(s) and kind, technically competent clinical staff are highlighted among others, such as respectful maternity care, effective communication, or continuity of care [6].

The worldwide epidemic of the Severe Acute Respiratory Syndrome Coronavirus (SARS-CoV-2) caused COVID-19 to spread across Europe at the beginning of 2020 and led to a completely unprecedented response in health and societal systems. The first case of COVID-19 in the Czech Republic was identified at the beginning of March 2020, and the disease began to spread. As a result, the Czech government announced a state of emergency on 12 March 2020 for 30 days [7], fundamentally limiting the social and economic life of the country. The state of emergency was repeatedly prolonged until 17 May 2020 [8]. As a part of this situation, the Ministry of Health of the Czech Republic prohibited visits to inpatient facilities in the interest of public health [9]. This extraordinary measure also included the presence of a partner or a close person during labor and birth. As a result, from 18 March to 16 April 2020, women in the Czech Republic gave birth without the presence of the child´s father or another partner/person. This was due to concerns about the transmission of infection from persons accompanying the woman at delivery to the staff of the birthing facilities. Thus, the right of the father to be present at the birth of his child was temporarily overridden by the protection of public health [10]. This was a unique situation in the Czech Republic that was unprecedented for many decades. Pregnant women form a significant and relatively large part of the population that is more vulnerable to mental health changes than the general population, and thus, they were at risk of becoming victims of measures that were epidemiologically justified but restrictive and highly stressful in their consequences.

Research to date has shown that this period was associated with negative outcomes on physical and psychological health, as well as changes in the use of health care, including fear of visiting medical facilities among the population [11]. A review published by Connor et al. [12] found that during the COVID-19 pandemic, women, in general, were exposed to a significant worsening of multifactorial stress. Pregnant women, whose changes in behavior and mental health have often been described, were monitored in particular during this period of increased psychological strain [13]. Caparros–Gonzalez et al. [14] stated that stress and depression in connection with pregnancy and labor during the COVID-19 pandemic were found worldwide, regardless of geographic or cultural factors.

Preis et al. [15] studied two types of stress situations associated with labor during the first few months of the pandemic. They distinguished the stress that is typically associated with the process of labor, so-called preparedness stress, and the stress from the fear of possible infection during the COVID-19 pandemic, so-called perinatal infection stress. Based on a questionnaire, the authors found that almost 18% of women reported a high level of both types of stress and that the perinatal infection stress was higher than the preparedness stress. In another questionnaire study of 336 pregnant women in Israel in March 2020, Taubman–Ben-Ari et al. [16] described a high level of stress in all areas of life, including worry about modes of travel, time spent in public places, fear of infecting family or the fetus, as well as fear of antenatal examinations and the birth itself. Karavadra et al. [17] used a thematic qualitative study to assess the experiences of pregnant women in association with COVID-19 and access to health care in Great Britain. The women reported fears about changes in the availability of services, including supportive care and the lack of the father during birth. The authors found that the worry of study participants about the lack of a partner during labor and birth included fears of being alone during birth if complications should arise. Surprisingly, the women were not worried about becoming infected and developing COVID-19 themselves but were worried about possible infections in their children. A high level of stress among pregnant women was also found in an Italian study [18]. Other studies [19] found that there were changes in the care provided to pregnant women during the COVID-19 pandemic. Such changes included both the presence of a partner during the birth itself (i.e., during the birth and one hour after), as well as limited rights of women for longer-term accompaniment, for example, during examinations, as was the case in Great Britain, France, and The Netherlands [20]. A more recent study on pregnant women during the COVID-19 pandemic in Denmark [21] showed that the greatest concern expressed in the survey was the risk of giving birth without the partner being present due to restrictions from the authorities.

Changes to the provided care, including limiting the presence of a partner during birth, are assumed to be an important stress factor for pregnant women, but robust evidence is lacking on how this influenced the health of pregnant women and their newborns. Most of the literature has focused on mothers that were ill with COVID-19, healthy newborns that were COVID-19 positive, as well as comparisons of births during the COVID-19 pandemic and previous epidemics such as SARS, which had markedly different conditions. Considering the urgency and unexpectedness of this issue, most published information has significant limitations, but it is, nevertheless, clear that the limitation or complete prevention of the presence of a partner during birth is considered to be a stress factor that negatively influences the psychological and physical health of pregnant women, even though information on the impact of this situation on the health of mothers and their newborns is largely lacking.

Although psychological stress is considered a causative factor in pre-term births, another Danish study found, on the basis of a national register, rather a decline in the incidence of extremely pre-term births (<28 completed weeks of gestation) during the first wave of lockdowns. Such a lowering of extremely pre-term births is highly positive since this decreases both perinatal and postnatal mortality and morbidity. The authors of that study speculated that the reasons for this decline might have been due to the fact that the lockdown led to dramatic changes in lifestyle, including lower physical activity in pregnant women, as well as the influence of hygienic practices leading to lower exposure to infectious agents in general, since infections are among the major factors inducing pre-term births [22].

The aim of this study is to assess the influence of the extraordinary and unique short-term nationwide ban on the presence of a partner or close person during labor in the context of societal stress caused by the COVID-19 pandemic on perinatal indicators in the Czech Republic from March to April 2020.

## 2. Materials and Methods

### 2.1. Data Collection

To obtain detailed information on possible perinatal complications caused by the ban on the presence of a partner during labor, we designed an observational study targeting all maternity facilities in the Czech Republic. Four weeks after the Ministry of Health lifted the ban and the situation was partially normalized, the researchers sent to all 88 maternity facilities in the country a questionnaire structured to assess basic perinatal indicators (binary or categorical absolute frequency data describing deliveries and their clinical characteristics) during the period from 18 March to 16 April 2020. For control, the same indicators were also collected for the equivalent period during 2019 (with no restrictions due to COVID-19). Replies (the head doctor or the head nurse of each maternity facility was responsible for data collection) were received from 34 facilities, but one was excluded from further analysis because its data was provided only for 2020. Thus, a total of 33 facilities were included in the study (a reply rate of 37.5%), covering 4805 births during the study period in 2019 and 4514 births in 2020. These numbers represented 51% of the total number of births in the Czech Republic during the periods studied. The issue of non-response bias is discussed in the limitations section.

### 2.2. Dissemination Strategy

The questionnaire was sent in electronic form with an accompanying letter by email on 15 May 2020 to all regional perinatologists in the Czech Republic (a traditional communication channel for the Czech Gynecological and Obstetrical Society, Prague, Czech Republic), who then forwarded the questionnaire to the heads of maternity facilities in their region. The cut-off date for returning the questionnaires was 31 May 2020.

### 2.3. Indicators Analyzed

The questionnaire was designed to monitor the total number of births, incidence of spontaneous pre-term births (<37 + 0 completed weeks of gestation), incidence of emergency and planned cesarean sections, number of inductions of labor, the use of analgesia during labor (including methods of systemic and/or regional analgesia), augmentation of labor with synthetic oxytocin, the average length of the first and second stages of labor, number of instrumental deliveries (forceps and/or vacuum extraction), the incidence of episiotomies (sorted by parity), the incidence of serious injuries during birth, and surgical procedures during the third stage of labor. Attention was given to postnatal adaptation of the newborn, monitoring the number of newborns with a 5-min Apgar score < 7, and pH of umbilical cord blood < 7.10. To obtain data on maternal blood loss due to peripartum bleeding, the use of supplementary blood products (packed red blood cells (PRBC), fresh frozen plasma (FFP), and fibrinogen substitution were monitored. In order to be comprehensive, we also included questions on the incidence of eclampsia, hysterectomies associated with birth, and maternal death.

### 2.4. Statistical Analysis

After receiving completed questionnaires from individual facilities, a data matrix was assembled. Data on basic indicators were available from all facilities. For other indicators, data were included only for those facilities that provided the information (sporadically, some data were missing as they are not routinely recorded in the respective facilities). Out of 51 indicators followed in total, data on 20 were provided by all 33 facilities, whereas data on only four indicators were provided by less than 29 facilities (non-pharmacological analgesia: 25; average length of the second stage of labor: 28; number of newborns with umbilical cord blood pH < 7.10: 27; the use of fresh frozen plasma: 27). All indicators, along with the number of facilities providing the respective data, are shown in Table 1. Pearson’s χ^2^ homogeneity test in 2 × 2 contingency tables was used to assess changes in individual indicator incidences between 2019 and 2020.

Incidence data were analyzed for the sum of all facilities in the Czech Republic (the nationwide perspective, see Table 1) as well as for each facility separately (discussed below if differed from the nationwide results). For indicators with low numbers of incidence, only combined values for the whole country were used. Tests were performed using Pearson’s χ^2^-test, which compared the incidence for the periods from 18 March to 1 April in 2019 and 2020. The indicator was valued as either a presence or absence using a two-by-two contingency table. On one occasion, a bigger table was used to test more options (4 types of birth).

## 3. Results

**Figure 1:**
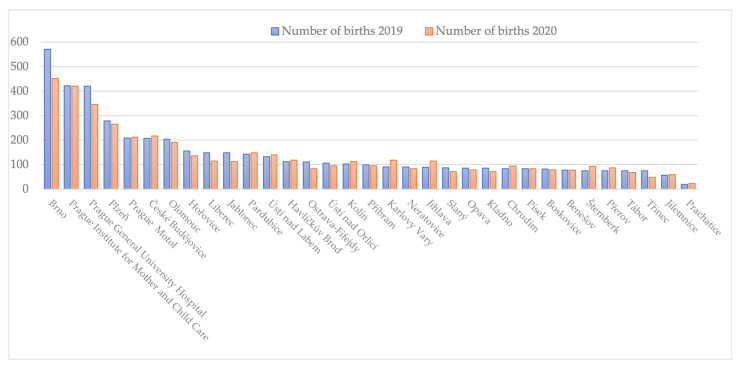
Births at maternity facilities, comparing the period from March 18th to April 16th in 2019 and 2020.

In April 2019, altogether, 9292 children were born in the Czech Republic, while 8859 were born in April 2020. This difference is in line with the inter-year variability (from 2011–2020, the average number of infants born in April is 8999 (95% CI: 8814, 9185)) [23]. The number of children born in the studied periods at individual facilities is shown in Figure 1. The facilities are listed according to the number of births in 2019. Figure 1 shows that the decline was mainly seen at the hospitals of FN Brno and VFN in Prague. Our dataset covers 4805 births in 2019 (representing 51.7% of all births in the Czech Republic during April 2019) and 4514 births in 2020 (51% of all births in April 2020).

Comparing the number of cesarean sections with the total number of births for the whole Czech Republic, the difference between 2019 and 2020 was not statistically significant (*p* = 0.536). This was also the case in most individual facilities, with the exception of an increase in cesarean section rate at Hořovice (*p* = 0.005), Jilemnice (*p* = 0.037), and Ústí nad Labem (*p* = 0.007), and a decrease in cesarean section rate at Přerov (*p* = 0.008). For a more detailed picture, we tested the changes in the mean of four values: vaginal full-term births, vaginal pre-term births, emergency cesarean sections, and planned cesarean sections (at facilities with a low number of pre-term births, only three values were tested, combining full-term and pre-term vaginal births; for three facilities, Hořovice, Prachatice, and Ústí nad Orlicí, numbers of pre-term vaginal births and planned cesarean sections were also too low to analyze them separately). From the national perspective, there was a statistically significant change (*p* = 0.030) caused mainly by the decline in emergency cesarean sections and an increase in planned cesarean sections. At individual facilities, statistically significant changes were found at Hořovice (*p* = 0.019)—an increase in emergency and planned cesarean sections and an associated decline in vaginal births; Pardubice (*p* = 0.025)—an increase in pre-term vaginal births; Přerov (*p* = 0.007)—a decline in emergency cesarean sections; Třinec (*p* = 0.026)—an increase in planned cesarean sections; and Ústí nad Labem (*p* = 0.002)—a decline in pre-term vaginal births and increase in emergency cesarean sections.

To compare the course of labor and delivery, we analyzed the incidence of extraction methods (using forceps or vacuum extraction) compared to the total number of vaginal births. Due to low numbers of such methods at individual facilities, only values from all facilities combined were analyzed. There was a statistically significant change in the increase in the use of these methods (*p* = 0.001). This was primarily due to an increase in the use of vacuum extraction, from 104 to 150 cases, accompanied by the decline in the total number of births (with the largest changes at the facilities of České Budějovice, Plzeň, and Olomouc). We also analyzed the incidence of induction of labor compared to the number of vaginal births and emergency cesarean sections. For all facilities combined, there was a statistically significant decline in the incidence of inductions (*p* = 0.016), mainly due to declines at the facilities at Karlovy Vary (*p* < 0.001) and Ústí nad Orlicí (*p* = 0.040); at other facilities, there were no significant changes (*p* > 0.05) or the numbers of inductions were too low to analyze (Jihlava, Prachatice).

As for the use of analgesia, there were highly significant differences among individual facilities. The results show that each obstetric facility used quite different procedures. The number of individual types of analgesia was compared to the total number of vaginal births. For all facilities combined, there was no significant difference found. However, this was caused by the averaging of different results from the facilities. At individual facilities, there were both significant increases as well as significant decreases in the types of analgesia used. For the categories “Non-pharmacological analgesia” and “No analgesia”, the use of these methods even ranged from “Always” to “Never” (non-pharmacological analgesia *p* = 0.595; systemic a. *p* = 0.964; regional a. *p* = 0.677; no analgesia *p* = 0.934).

The incidence of the use of episiotomy was compared with total vaginal births. Data were available for all participating facilities except for Plzeň, which provided only total values without specifying the parity of mothers (primiparous and multiparous mothers). Nationwide, there was a statistically significant decline (*p* = 0.022), which was caused by values for multiparous mothers (*p* < 0.001) rather than for primiparous mothers (*p* = 0.122). For individual facilities, there was a significant increase in the number of episiotomies found at Kolín (*p* = 0.05; i.e., at the border of significance), Písek—a significant increase for multiparous mothers (*p* = 0.047), and VFN Prague—a significant increase for primiparous mothers (*p* = 0.006), but a significant decline for multiparous mothers (*p* < 0.001) as well as in the total incidence (*p* = 0.001).

Analyzing the incidence of surgical procedures during the third stage of labor compared to the total number of vaginal births, there was a statistically significant difference between 2019 and 2020, similar to the incidence of third- and fourth-degree perineal tears compared to the total number of vaginal births. There was no statistically significant difference found in the number of newborns with a 5-min Apgar score < 7, the number of newborns with umbilical cord blood pH < 7.10, the use of supplementary blood products (PRBC, FFP), or the use of supplementary fibrinogen; all these indicators were compared to the total number of births.

Furthermore, there was no incidence of eclampsia reported by the hospitals for either 2019 or 2020 (data from Plzeň was lacking). Hysterectomy associated with birth was reported only in rare cases (2019: Olomouc, Pardubice, Třinec (3 cases in total), 2020: Šternberk, 2 × UPMD, VFN Prague (4 cases in total)). Two cases of peripartum maternal deaths were reported in 2020 (at Jihlava and VFN Prague), while there was no case in 2019.

## 4. Discussion

During the first wave of the COVID-19 pandemic in the Czech Republic, a nationwide ban was introduced that included a ban on the presence of a partner or other close person in maternity wards. This allowed us the opportunity to compare basic perinatological parameters during two comparable periods but differing in the presence of a birthing partner during labor and delivery. Given the size of the research sample, the results of the study can be considered nationally representative; 8859 children were born in the Czech Republic during the period studied in 2020 compared to 9292 a year earlier. This difference corresponded to the year-to-year fluctuation of the birthrate in the long term. There was no statistically significant change (*p* = 0.536) in the number of cesarean sections over the periods studied. Regarding extraction methods, there was a statistically significant increase in the frequency of these methods (*p* = 0.001); however, the change was due to the situation in two facilities only, and the reason was not specified by these facilities. As for analgesia, there was no statistically significant change during the observation periods. Overall, there was a statistically significant reduction in the length of the first stage of labor and a decrease in the incidence of episiotomies (*p* = 0.022), which was due to values in multiparous women (*p* < 0.001), while there was no statistically significant change in primiparous women (*p* = 0.122). The explanation for the discrepancy in the incidence of episiotomies between primiparous and multiparous women could not be sufficiently clarified within the study design, nor was it a primary objective. There was no statistically significant difference in the frequency of surgical intervention in the third stage of labor analyzed in relation to the total number of vaginal deliveries. Except for the increase in vacuum extraction, no difference was found in any of the perinatological parameters studied.

The impact of stress in the context of the COVID-19 pandemic on mothers and births has been discussed by Matvienko-Sikar et al. [24], who described the impact of stress on mothers and called for essential psychological support for mothers by healthcare professionals. However, they did not report a worsening in maternal outcomes due to COVID-19, in agreement with the conclusions of Elshafeey et al. [25]. Gausman and Langer [26] highlighted the disproportionate impact of the pandemic on women in the context of COVID-19, citing, among other factors, the fact that women give birth in pandemic settings without social support. A more recent study by Harrison et al. [27] found negative relationships between perceived social support and depression and anxiety in a sample of women who were pregnant during the COVID-19 pandemic, indicating that women with lower levels of perceived support experienced more depression and anxiety symptoms, in alignment with research conducted prior to the pandemic. Mollard and Wittmaack [28] suggested that pandemic-related changes to maternity care practices may have impacted birthing women’s perceptions of safety and support in the hospital environment and affected symptoms of stress. On the one hand, the available literature shows that there is no evidence of poorer obstetric outcomes during the COVID-19 pandemic; however, the literature also shows that maternal stress has been substantial during the pandemic and that a responsive and understanding approach by health care professionals is needed.

In the Czech Republic, the ban on a partner during childbirth provoked a strong wave of emotions and negative reactions across the country. The presence or absence of the father during labor and delivery of their children became a frequent topic in both traditional and new types of media. All this multiplied the antenatal stress levels in women who were due to give birth during this period. Even the Cochrane review [3] was used as an argument; however, the most recent review on continuous support for women during childbirth from 2017 did not take into consideration issues such as the COVID-19 pandemic [29] and the importance of continuous support has never been neglected. Despite the epidemiologically caused absence of a close person at birth, continuous midwifery support was provided to every woman under all circumstances in all facilities. Therefore, by inducing hostility and even panic in society, an emotionally strained atmosphere was created in the maternity sector, which may have led to fears of vulnerability and distrust in the practical and psychological support of obstetric teams. After the stabilization of the epidemiological situation in the Czech Republic and after the expansion of expert knowledge on the biological effects of SARS-CoV-2, the strict ban on the presence of the father or another non-hospital birthing partner began to be abolished after four weeks [10].

The number of completed questionnaires returned (34/88) clearly demonstrates that healthcare professionals put a high emphasis on the issue of partners’ participation in childbirth. However, the perinatological results obtained in the questionnaire study are, in some respects, rather surprising. Apart from the increase in the frequency of extraction methods, no perinatological results were observed that would demonstrate a negative impact on perinatological outcomes in terms of the absence of a partner or other close person during childbirth, particularly taking into consideration the impact of ante/intrapartum stress, which was extreme due to the measures mentioned.

The results of this study are unique because conducting a randomized control trial on the matter of the absence of a partner at birth is otherwise impossible for ethical reasons, and a recurrence of the national ban on the presence of a partner or close person at birth is very unlikely to occur in the foreseeable future.

### Limitations of the Study

The first limitation of our work is that our study has not focused on the possible multifactorial subjective psychological impact on labor. We focused primarily on objective clinical indicators.

Another limitation is the non-response bias. The data cover the biggest maternity hospitals in the country but exclude data from some facilities. However, data were provided by facilities from 12 out of 14 administrative regions of the Czech Republic (the two missing are the Hradec Kralove Region and Zlín Region) and include all types of maternity hospitals ranging from university hospitals to regional hospitals to municipal or private maternity facilities. We conducted our analysis not only based on nationwide data but also for each participating maternity facility individually, and if the results differed, we discussed them in the text. Thus, all trends could be captured, and the results are quite representative of the Czech Republic.

The time period in which the data was obtained was also relatively short. A total of 8 of the 12 perinatology intensive care centers and 9 of the 13 perinatology intermediate care facilities returned completed questionnaires. 

A final limitation is an inability to assess the impact of this measure in comparison with other epidemiological measures in the context of the first wave of the COVID-19 pandemic in the Czech Republic.

The impact of the absence of a close person at birth in other countries where their presence is not part of the standard approach to childbirth, whether for cultural, religious, economic, or other reasons, is not reflected in our work and requires a considered approach.

## 5. Conclusions

This study offers first insights into a situation where the presence of a person other than healthcare professionals was not possible during labor and at birth for a limited period. Women giving birth alone (i.e., without a non-hospital birthing partner) due to the first wave of the COVID-19 pandemic did not show different perinatological outcomes compared to the same period in 2019. During the period of specific government-mandated anti-epidemic measures, we did not observe changes in the incidences of cesarean sections or other types of operative deliveries, or increases in pre-term births, the duration of the second stage of labor, the use of synthetic oxytocin for augmentation, the need for the administration of analgesics, the incidence of fetal hypoxia, the incidence of episiotomies or other birth injuries, blood loss, or neonatal complications requiring special care. There was only a local increase in the number of vacuum extractions. There were significant decreases in the number of inductions of labor, the duration of the first stage of labor, and episiotomies in multiparous women. In terms of the perinatological parameters assessed, the absence of a partner or other close person when giving birth during the COVID-19 pandemic did not cause a deterioration in the quality of perinatal care provided.

## Figures and Tables

**Table 1:** A summary of results of the test of homogeneity between the 2019 and 2020 results from the nationwide perspective (summary data of all participating maternity facilities).

Indicator	Average Number of Births	Vaginal Births vs. Caesarean Sections	Vaginal Full Term vs. Pre-Term	Non-Pharma Analgesia (Yes-No)	Systemic Analgesia (Yes-No)	Regional Analgesia (Yes-No)	No Analgesia (Yes-No)	Oxytocin (Yes-No)	Total Episiotomy (Yes-No)	Episiotomy— Primiparous Mothers (Yes-No)	Episiotomy—Multiparous Mothers (Yes-No)
n ^a^	33	33	33	25	32	33	29	31	33	32	32
*p*-value ^b^	0.030 *	0.536	0.522	0.595	0.964	0.677	0.934	0.441	0.022 *	0.122	<0.001 **
Indicator	Induced births (yes-no)	Vacuum extraction (yes-no)	Forceps (yes-no)	Surgery in third stage of labor (yes-no)	3rd or 4th degree perin. tears (yes-no)	5-min Apgar score < 7 (yes-no)	Umbilical cord blood pH < 7.17 (yes-no)	Use of PRBC (yes-no)	Use of FFP (yes-no)	Fibrinogen (yes-no)	
n ^a^	33	32	32	32	32	32	27	30	27	30	
*p*-value ^b^	0.016 *	0.001 **	0.535	0.581	0.573	0.837	0.997	0.417	0.270	0.141	

^a^ Number of facilities reporting this data; ^b^ results of Pearson’s χ^2^ homogeneity test. * Statistical significance α = 0.05; ** statistical significance α = 0.01.

## Data Availability

The dataset used and analyzed during the current study is available from the corresponding author upon making official request addressed to the Department of Obstetrics and Gynecology, First Faculty of Medicine, Charles University and General University Hospital in Prague.
